# JNJ-77242113, a highly potent, selective peptide targeting the IL-23 receptor, provides robust IL-23 pathway inhibition upon oral dosing in rats and humans

**DOI:** 10.1038/s41598-024-67371-5

**Published:** 2024-07-30

**Authors:** Anne M. Fourie, Xiaoli Cheng, Leon Chang, Carrie Greving, Xinyi Li, Beverly Knight, David Polidori, Aaron Patrick, Trpta Bains, Ruth Steele, Samantha J. Allen, Raymond J. Patch, Chengzao Sun, Sandeep Somani, Ashok Bhandari, David Liu, Keith Huie, Shu Li, Michael A. Rodriguez, Xiaohua Xue, Arun Kannan, Teddy Kosoglou, Jonathan P. Sherlock, Jennifer Towne, M. Claire Holland, Nishit B. Modi

**Affiliations:** 1 Janssen Research & Development, LLC, La Jolla, CA USA; 2 Protagonist Therapeutics, Newark, CA USA; 3 Janssen Research & Development, LLC, Spring House, PA USA

**Keywords:** Interleukins, Clinical pharmacology

## Abstract

The interleukin (IL)-23 pathway is a pathogenic driver in psoriasis, psoriatic arthritis, and inflammatory bowel disease. Currently, no oral therapeutics selectively target this pathway. JNJ-77242113 is a peptide targeting the IL-23 receptor with high affinity (K_D_: 7.1 pM). In human cells, JNJ-77242113 potently and selectively inhibited proximal IL-23 signaling (IC_50_: 5.6 pM) without impacting IL-12 signaling. JNJ-77242113 inhibited IL-23–induced interferon (IFN)γ production in NK cells, and in blood from healthy donors and psoriasis patients (IC_50_: 18.4, 11 and 9 pM, respectively). In a rat trinitrobenzene sulfonic acid-induced colitis model, oral JNJ-77242113 attenuated disease parameters at doses ≥ 0.3 mg/kg/day. Pharmacologic activity beyond the gastrointestinal tract was also demonstrated. In blood from rats receiving oral JNJ-77242113, dose-dependent inhibition of ex vivo IL-23–stimulated IL-17A production was observed. In an IL-23–induced rat skin inflammation model, JNJ-77242113 inhibited IL-23–induced skin thickening and IL-17A, -17F and -22 gene induction. Oral dosing of JNJ-77242113 in healthy human volunteers inhibited ex vivo IL-23–stimulated IFNγ production in whole blood. Thus, JNJ-77242113 provided selective, systemic IL-23 pathway inhibition in preclinical models which translated to pharmacodynamic activity in healthy human volunteers, supporting the potential for JNJ-77242113 as a selective oral therapy for IL-23–driven immune-mediated diseases.

## Introduction

Interleukin (IL)-23 is a disulfide-linked heterodimer composed of two subunits: IL-23p19 and IL-12/23p40^1^. The IL-23 receptor is also comprised of two subunits: IL-23R, which binds exclusively to IL-23p19, and IL-12Rβ1, which binds to IL-23 or IL-12 through a shared p40 subunit^1^. Binding of IL-23 to its receptor results in the phosphorylation of janus kinase 2 (JAK2) and tyrosine kinase 2 (TYK2) and the subsequent phosphorylation of signal transducer and activator of transcription (STAT) proteins which, in turn, promotes expression of downstream cytokines including IL-17A, IL-17F, IL-22, and interferon (IFN)γ^1–3^. Although multiple STAT proteins are phosphorylated in response to IL-23 signaling, the biological effects of IL-23 activity are thought to be mediated predominantly through STAT3^4^.

IL-23 signaling plays a key role in immune-mediated inflammatory diseases, such as psoriasis, psoriatic arthritis, and inflammatory bowel disease (IBD)^2,5–8^. In a mouse model of T-cell–mediated colitis, genetic ablation of IL-23p19 eliminated intestinal inflammation^9^. Subsequent studies in murine models confirmed a central role for IL-23 in colitis^10–14^. In another study, intradermal injection of IL-23 induced induration, erythema, and epidermal hyperplasia in wild-type mice, features characteristic of psoriatic lesions in humans^15^. Treatment of a transgenic mouse model of psoriasis with anti–IL-23 antibodies ameliorated pathologic features, such as epidermal hyperplasia, to a greater extent than treatment with antibodies targeting IL-17^16^. In humans, polymorphisms in IL-23R are directly associated with risk of developing certain inflammatory diseases^17–20^. In particular, a loss-of-function mutation (R381Q) in IL-23R, which results in decreased downstream IL-17 production and reduced STAT3 activation, is protective in psoriasis and IBD^17,19,21^, providing the basis for IL-23R antagonism as a therapeutic approach in these diseases. Data supporting the critical pathogenic role of IL-23 in inflammatory diseases has led to development of therapies that inhibit the IL-23 pathway and IL-23–targeted biologics have been clinically validated in patient populations with psoriatic skin and joint inflammation and in IBD^5,22–25^.

JNJ-77242113 (formerly PN-235) is a potent peptide targeting IL-23R and the first selective IL-23 pathway modulator that can be delivered orally. In a recent phase 2 study conducted in patients with moderate-to-severe psoriasis, JNJ-77242113 met its primary clinical efficacy endpoint^26^. Here, we characterize the pharmacology, pharmacodynamics, and safety of JNJ-77242113 in biophysical assays, human immune cells, preclinical rodent models, and in a first-in-human clinical trial. JNJ-77242113 provided selective IL-23 pathway inhibition in vitro and in preclinical models; this activity successfully translated to pharmacodynamic activity in healthy human volunteers, indicating promise as a future oral therapy for IL-23–driven diseases.

## Results

### Molecular properties of JNJ-77242113

**Figure 1:**
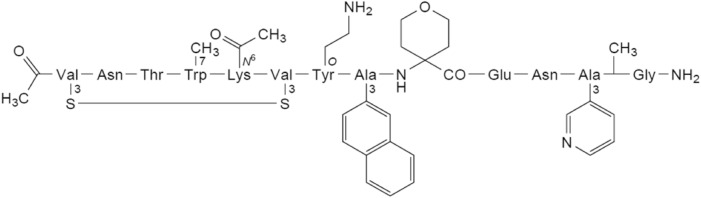
Chemical structure of JNJ-77242113 (S^3,1^,S^3,6^-cyclo[*N*-acetyl-3-sulfanyl-L-valyl-L-asparaginyl-L-threonyl-7-methyl-L-tryptophyl-*N*^6^-acetyl-L-lysyl-3-sulfanyl-L-valyl-*O*-(2-aminoethyl)-L-tyrosyl-3-(naphthalen-2-yl)-L-alanyl-4-aminooxan-4-carbonyl-L-α-glutamyl-L-asparaginyl-3-(pyridin-3-yl)-L-alanyl-*N*^2^-methylglycinamide]).

JNJ-77242113 is a chemically-synthesized macrocyclic peptide with the structure shown in Fig. 1 and a molecular weight of 1898.19 g/mol.

### In vitro pharmacology

As measured by surface plasmon resonance (SPR), JNJ-77242113 bound to the human IL-23R extracellular domain (ECD) with high affinity, with a mean dissociation constant (K_D_) of 7.1 pM at 37 °C (Table 1 and Fig. S1A). Similar binding affinity was determined for the rat IL-23R ECD (K_D_ of 17.5 pM at 37 °C; Table 1 and Fig. S1B).

**Table 1:** IL-23R binding affinity and potency of JNJ-77242113 against IL-23–mediated responses in cells.

In vitro protein/cells	Binding affinity/IL-23–induced endpoint	JNJ-77242113 K_D_ or IC_50_ (pM)	K_D_/IC_50_ range^c^	n^d^
Human IL-23R ECD (SPR in vitro)	Binding affinity (K_D_)	7.1 ± 2.5	4–10	5
Human PBMC	IL-23–induced STAT3 phosphorylation	5.6 ± 1.2	4.3–6.6	3
Human PBMC	IL-12–induced STAT4 phosphorylation^a^	> 2,000,000	–	2
Human NK cells	IL-23–induced IFNγ production	18.4 ± 6.2	12.4–28.3	5
Human (healthy) whole blood	IL-23–induced IFNγ production	11^b^	4–91	15
Human (psoriasis) whole blood	IL-23–induced IFNγ production	9^b^	0.5–35	4
Rat IL-23R ECD (SPR in vitro)	Binding affinity (K_D_)	17.5 ± 7.8	12–23	2
Rat whole blood	IL-23–induced IL-17A production (20 ng/mL IL-23)	250 ± 62	160–340	6
Rat whole blood	IL-23–induced IL-17A production (4 ng/mL IL-23)	54 ± 34	12–110	8

*ECD* extracellular domain, *IC*_50_ 50% inhibitory concentration, *IFN* interferon, *IL* interleukin, *K*_D_ disassociation constant, *NK* natural killer, *PBMC* peripheral blood mononuclear cells, *SPR* surface plasmon resonance, *STAT* signal transducer and activator of transcription.

^a^Impact on IL-12 signaling in same PBMCs to assess selectivity.

^b^Values for human whole blood IC_50_s are median values; all others are mean values.

^c^Range indicates minimum and maximum values obtained across independent experiments.

^d^Number of independent experiments, n.

**Figure 2:**
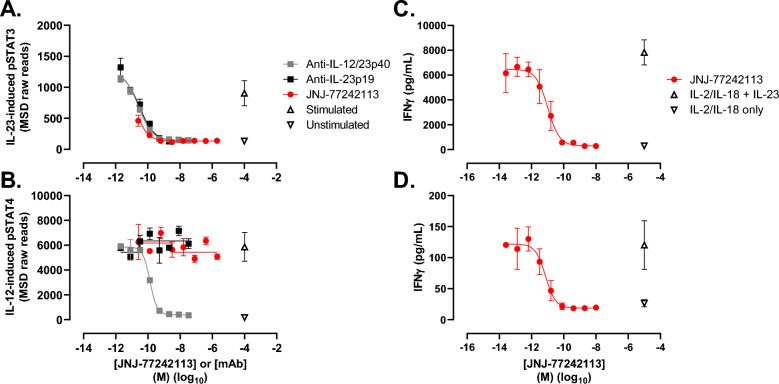
JNJ-77242113 inhibited IL-23 receptor–mediated proximal signaling and downstream cytokine production in immune cells. IL-23–induced pSTAT3 (**A**) and (**B**) IL-12–induced pSTAT4 in human PBMCs. Concentration-dependent inhibition of IFNγ production in whole blood from (**C**) a healthy donor and (**D**) a donor with psoriasis. Panels (**A**) and (**B**) show data from one donor that is representative of 3 and 2 experiments, respectively. Panels (**C**) and (**D**) show data from one donor that is representative of data from 15 and 4 donors, respectively (see Table 1 for data summary). Each point represents the mean of duplicate values (**A**,**B**) or triplicate values (**C**,**D**); error bars denote SD. An arbitrary concentration value on the log(x) axis was used to plot mean values for the unstimulated or IL-23/IL-12–stimulated controls. *IFN* interferon, *IL* interleukin, *MSD* Meso Scale Discovery, *PBMC* peripheral blood mononuclear cells, *pSTAT* phosphorylated signal transducer and activator of transcription, *SD* standard deviation.

The potency and selectivity of JNJ-77242113 in inhibiting IL-23–induced proximal signaling and downstream cytokine production were evaluated in human immune cells and whole blood (Table 1). In human peripheral blood mononuclear cells (PBMCs), JNJ-77242113 inhibited IL-23–induced STAT3 phosphorylation in a concentration-dependent manner with a half-maximal inhibitory concentration (IC_50_) of 5.6 ± 1.2 pM (IC_50_ ± standard deviation [SD]; Fig. 2A). IL-23–induced STAT3 phosphorylation, but not IL-12–induced STAT4 phosphorylation, was inhibited by JNJ-77242113 in a concentration-dependent manner, indicating selective inhibition of IL-23–induced signaling (Fig. 2B). Similar selective IL-23 inhibition was observed for an anti–IL-23p19 antibody, while an anti–IL-23p40 antibody inhibited signaling downstream of both IL-23 and IL-12 by targeting the common p40 subunit (Fig. 2A,B). Consistent selectivity results were obtained for JNJ-77242113, and the anti–IL-23p19 and p40 antibodies, when IL-23–induced pSTAT4 and IL-12–induced pSTAT3 were evaluated (data not shown). Concentration-dependent inhibition of IL-23–induced STAT3 phosphorylation by JNJ-77242113 in human diffuse large-cell B-lymphoma (DB) cells was investigated at different concentrations of IL-23 to determine the mode of inhibition. Analysis of these data indicated that JNJ-77242113 acted as a simple competitive antagonist of IL-23R in DB cells (Fig. S2).

### In vitro inhibition of IFNγ production in human natural killer (NK) cells and whole blood from healthy volunteers and psoriasis patients

Inhibition of downstream cytokine production was studied in human NK cells and human whole blood. JNJ-77242113 inhibited IL-23–stimulated IFNγ production in the presence of IL-2 and IL-18, with an IC_50_ value of 18.4 ± 6.2 pM in human NK cells (Table 1). Whole blood from healthy volunteers or psoriasis patients was pretreated in vitro with JNJ-77242113, then stimulated with IL-23 in the presence of IL-2 and IL-18. JNJ-77242113 inhibited IL-23–induced IFNγ production in a concentration-dependent manner in whole blood from healthy donors, with a median IC_50_ value of 11 pM (range: 4–91 pM; n = 15 donors; Fig. 2C; Table 1). In blood from donors with psoriasis (n = 4), JNJ-77242113 inhibited IL-23–induced IFNγ production with a median IC_50_ value of 9 pM (range: 0.5–35 pM; Fig. 2D; Table 1), indicating an inhibitory potency similar to that observed in blood from healthy volunteers.

To further characterize the specificity of JNJ-77242113 for IL-23R, a panel of potential secondary pharmacodynamic targets (including receptors, ion channels, enzymes, and transporters) was screened for inhibition or activation by JNJ-77242113. Inhibition/activation was < 50% for all targets in the presence of 10 μM JNJ-77242113 (Table S1), further confirming the selectivity of JNJ-77242113 for IL-23R.

### In vivo pharmacology

#### Rat trinitrobenzene sulfonic acid (TNBS)-induced colitis model

**Figure 3:**
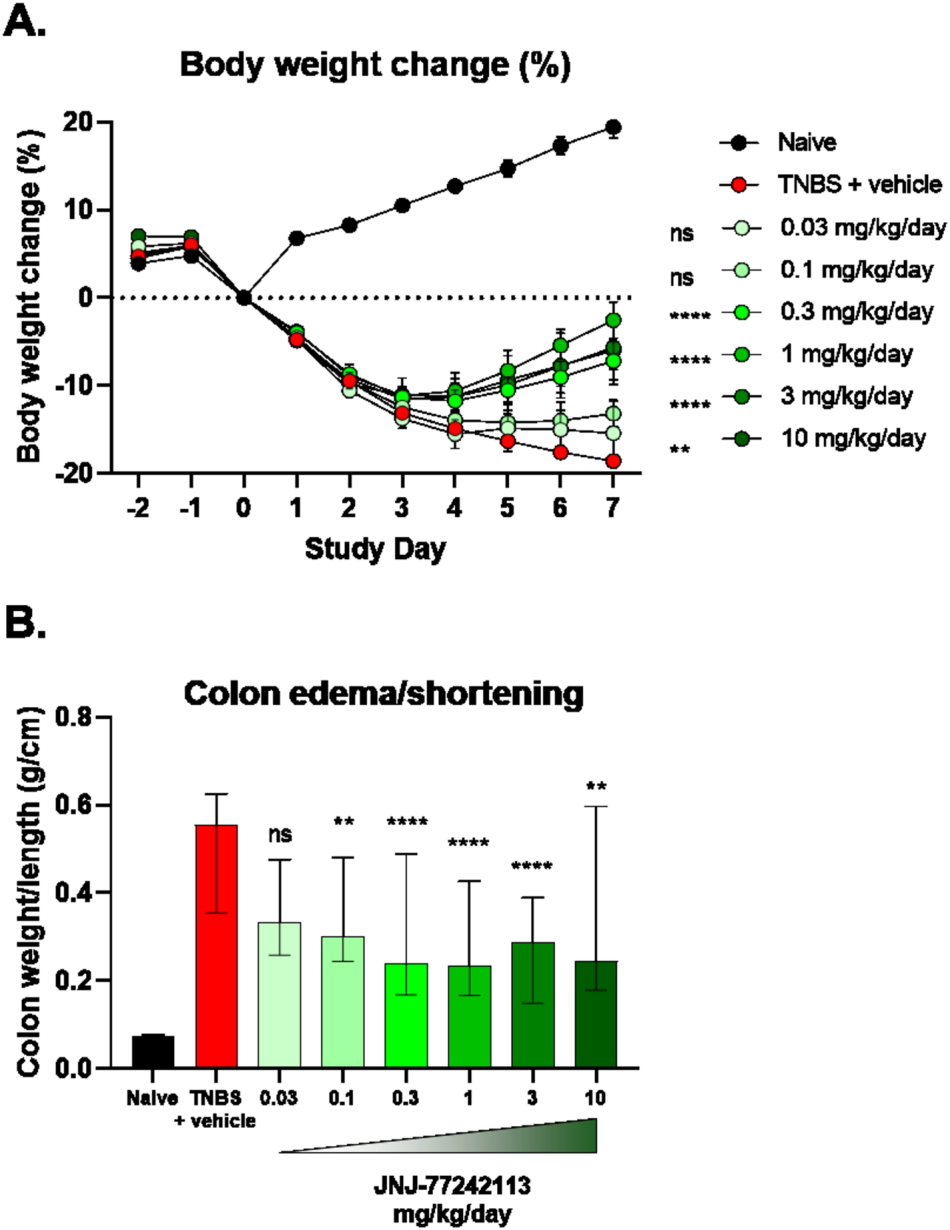
Treatment with JNJ-77242113 protected against body weight loss and signs of inflammation in a TNBS-induced colitis rat model. (**A**) Mean percent change in body weight from baseline over time (error bars represent SEM; **P < 0.001, ****P < 0.0001), (**B**) colon weight/length ratio at Day 7 (**P < 0.01, ****P < 0.0001). Bars represent medians and error bars denote interquartile ranges. n = 10 per dose group per experiment; data from up to 3 experiments were combined. *ns* not significant, *SEM* standard error of the mean, *TNBS* trinitrobenzene sulfonic acid.

Rat pharmacokinetic and tissue distribution studies following oral dosing of JNJ-77242113 indicated low oral bioavailability (data not shown), which is typical for oral peptides^27^, but detectable plasma concentrations and significantly higher exposures in intestinal tissues, mucus, mucosa, and colon fecal content (Table S2). Therefore, the in vivo activity of JNJ-77242113 was evaluated in a rat model of TNBS-induced colitis. JNJ-77242113 concentrations in colon tissue and colon content increased with increasing doses (Table S3), exceeding the in vitro IC_50_ in rat whole blood (Table 1). While rats that received rectal instillation of TNBS plus vehicle demonstrated marked weight loss compared to TNBS-naïve animals, oral treatment with JNJ-77242113 attenuated TNBS-induced weight loss (Fig. 3A). By Day 7, doses of 0.3, 1, 3, and 10 mg/kg/day provided significant treatment effects on body-weight loss (*P* < 0.001) versus the TNBS plus vehicle group.

TNBS-induced colitis is characterized by colon edema and shortening, resulting in an increased colon weight/length ratio. Compared to the TNBS plus vehicle group, TNBS-exposed rats treated with JNJ-77242113 at doses of 0.1, 0.3, 1, 3, and 10 mg/kg/day showed significantly reduced colon weight/length ratios (*P* < 0.01; Fig. 3B).

#### Rat pharmacokinetic-pharmacodynamic model of systemic activity

**Figure 4:**
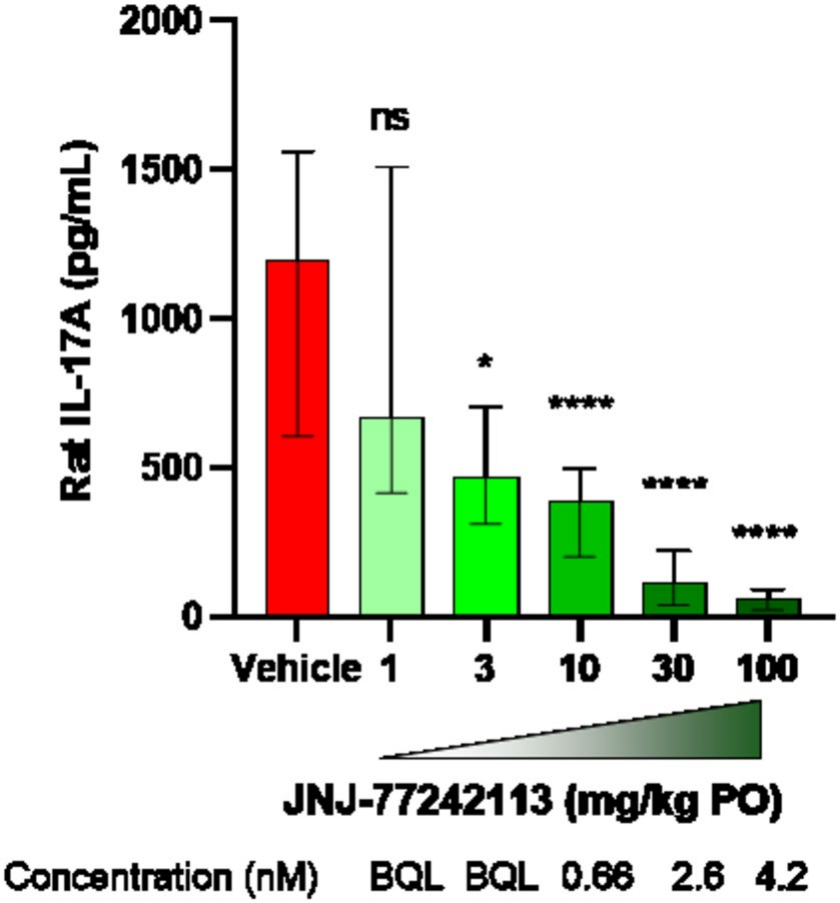
Inhibition of ex vivo IL-23–stimulated IL-17A production in rat whole blood following oral dosing with JNJ-77242113. IL-17A levels produced in whole blood samples (diluted 5 × in media) after addition of 4 ng/mL IL-1β were subtracted from IL-17A levels produced with 20 ng/mL IL-23 and 4 ng/mL IL-1β. Doses below 1 mg/kg (not shown; 0.03, 0.1, and 0.3 mg/kg) showed no significant difference from vehicle control. Bars represent medians and error bars denote interquartile ranges. Data from 5 experiments were combined. Plasma samples from each animal were analyzed using an LC–MS/MS method and dilution adjusted to report concentrations of JNJ-77242113 in the assay. *IL* interleukin, *LC–MS/MS* liquid chromatography tandem mass spectrophotometry, *ns* not significant, *PO* orally. *P < 0.05, ****P < 0.0001.

Although exposures upon oral dosing were highest in intestinal tissues (Table S2), the potential for systemic pharmacodynamic activity was also investigated in a rat pharmacokinetic-pharmacodynamic model. JNJ-77242113 was tested in vitro in a rat whole blood assay in which IL-23–induced IL-17A production in the presence of IL-1β was measured. In this assay, JNJ-77242113 dose-dependently inhibited IL-17A production, in response to stimulation with 4 and 20 ng/mL IL-23, at IC_50_ values of 54 ± 34 pM and 250 ± 62 pM, respectively (Table 1). The higher IC_50_ values at higher IL-23 concentrations were consistent with JNJ-77242113 acting as a competitive antagonist of rat IL-23R. To study the systemic pharmacodynamic effects of JNJ-77242113, rats were given different oral doses of JNJ-77242113, after which blood was drawn and stimulated ex vivo with IL-23 and IL-1β. The resulting IL-17A production was inhibited in a dose-dependent manner, with JNJ-77242113 doses of ≥ 3 mg/kg demonstrating significant effects (Fig. 4). Exposure–response relationships from in vivo dosing were consistent with the IC_50_ values observed in vitro in rat whole blood experiments (Table 1). Exposures from in vivo doses less than 10 mg/kg were below the limit of quantification. The mean dilution-adjusted exposure of 660 pM at 2 h (T_max_) in the 10 mg/kg group exceeded the IC_50_ value (250 pM) from the in vitro rat whole blood experiments and was, therefore, consistent with the > 50% inhibition of IL-17A production in that dose group. The dilution-adjusted exposures in the 30 and 300 mg/kg groups were greater than the in vitro IC_90_ values and, thus, consistent with maximal levels of inhibition observed in these groups.

#### Rat IL-23–induced skin inflammation model

**Figure 5:**
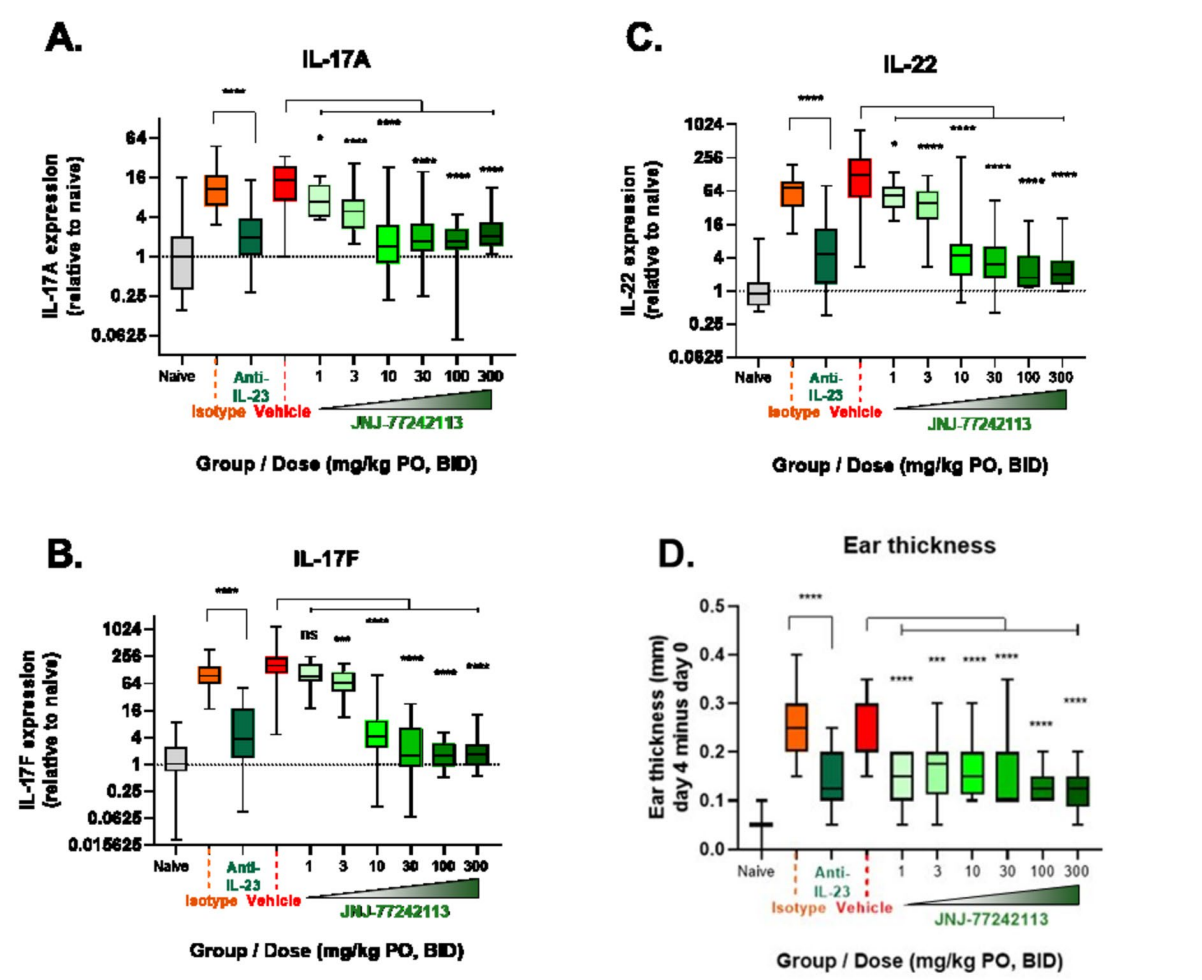
In a rat skin inflammation model, oral JNJ-77242113 showed systemic pharmacodynamic activity. Inhibition of IL-23 stimulated (**A**) IL-17A, (**B**) IL-17F, and (**C**) IL-22 expression in rat skin. (**D**) Change in ear thickness (Day 4–Day 0) was ameliorated by oral pretreatment with JNJ-77242113. Boxes show medians and interquartile ranges and error bars denote minima and maxima. n = 10 per dose group or n = 5 for vehicle groups per experiment; data from up to 3 experiments were combined. *BID* twice daily, *IL* interleukin, *ns* not significant, *PO* orally. *P < 0.05, ***P < 0.001, ****P < 0.0001.

The systemic activity observed in the rat whole blood model prompted further testing of tissue-specific activity of orally dosed JNJ-77242113 beyond the intestines. In a rat skin inflammation model, intradermal injection of IL-23 into the right ear dramatically increased expression of downstream genes, including those encoding IL-17A, IL-17F, and IL-22 (greater than 16-, 100- and 100-fold, respectively; Fig. 5A–C) compared with intradermal injection of saline. Orally dosed JNJ-77242113 prevented IL-23–induced upregulation of IL-17A and IL-22 at all doses and of IL-17F at all except the lowest dose (1 mg/kg twice daily [BID]; Fig. 5A–C). At doses ≥ 10 mg/kg BID, the extent to which JNJ-77242113 reduced IL-17A, IL-17F, and IL-22 gene expression was equivalent to or greater than that of an anti–IL-23 monoclonal antibody (4 mg/kg on Day -1 and Day 3, by intraperitoneal injection). Injection with IL-23 resulted in an increase in skin thickness, reaching an average of approximately 0.24 mm by Day 4. In comparison, IL-23–induced skin thickness was significantly lower on Days 3 and 4 in rats receiving oral JNJ-77242113 at doses of 1, 3, 10, 30, 100, and 300 mg/kg BID (Fig. 5D), outcomes consistent with those achieved with an anti–IL-23 antibody. In plasma samples taken 16 h after the final dose was administered, JNJ-77242113 levels were below the limit of quantification at all doses, except 300 mg/kg BID (data not shown).

### JNJ-77242113 pharmacology in humans

#### First-in-human study

Based on data from preclinical pharmacology and toxicology studies, a first-in-human study of JNJ-77242113 was conducted. A total of 95 healthy participants were enrolled in the single ascending dose (SAD; Part 1) and multiple ascending dose (MAD; Part 2) arms of the study. In Part 1 of the study (SAD Cohorts), 39 participants received a single dose of JNJ-77242113 (n = 29) or placebo (n = 10). In Part 2 (MAD Cohorts), 56 participants were enrolled (JNJ-77242113: n = 44; placebo: n = 12). The study was completed by all participants in Part 1; 2 participants in Part 2 withdrew consent for personal reasons unrelated to safety or tolerability. The majority of participants were white males; age, weight, and body mass index were generally comparable across Parts 1 and 2 (Table S4).

**Figure 6:**
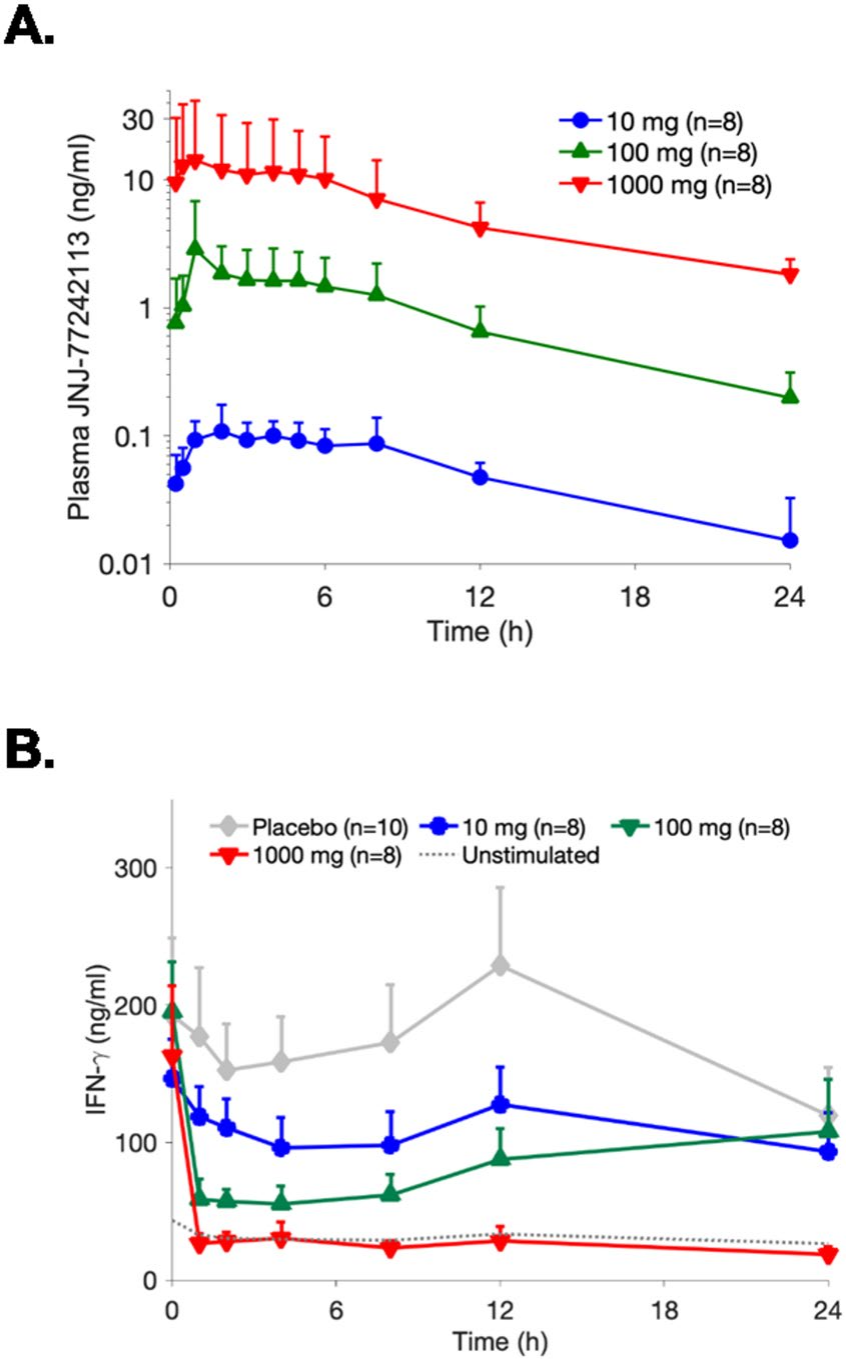
Pharmacokinetics and pharmacodynamics of orally administered JNJ-77242113 in a first-in-human study of healthy participants. (**A**) Mean plasma concentrations of JNJ-77242113 in healthy participants from Part 2 (Day 1 in multiple ascending dose cohorts). Error bars denote standard deviation. (**B**) Mean concentrations of IL-23–induced IFNγ in whole blood from participants after they had received a single oral dose of JNJ-77242113. Error bars denote standard error of the mean. *IFN* interferon, *IL* interleukin.

Dose-proportional plasma exposure of JNJ-77242113 was observed across a 100-fold dose range of 10 to 1000 mg (Fig. 6A), with an estimated terminal elimination half-life of 9–16 h. In Part 1, mean peak plasma concentrations (C_max_) increased in a slightly less than dose-proportional manner, whereas the area under the curve (AUC) increased proportionally with dose. In Part 2, C_max_ and AUC increased in a dose-proportional manner and a comparison of C_max_ and AUC values on Day 1 versus Day 10 indicated an accumulation of 0.7 to 1.6 for C_max_ and 0.9 to 1.5 for AUC with once daily dosing, consistent with an estimated half-life of 9–16 h. Whole blood from participants in Part 2 was evaluated for the pharmacodynamic effects of JNJ-77242113 in response to IL-23 stimulation. Ex vivo inhibition of IL-23–induced IFNγ production was observed after a single dose of 10 mg JNJ-77242113, compared with placebo, and was more robust with 1000 mg JNJ-77242113 (Fig. 6B), which showed complete inhibition of the IL-23–dependent IFNγ production for 24 h after dosing.

Across all cohorts, adverse events (AEs) were mild or moderate in severity. No serious AEs or deaths related to JNJ-77242113 were reported and there were no dose-related trends in the frequency of AEs. The frequency of treatment-related AEs was greater in the JNJ-77242113 groups compared with placebo (Part 1: 31% vs 20%, respectively; Part 2: 46% vs 25%, respectively). The most common AE was headache, reported in 10.3% and 13.6% of participants in Part 1 and Part 2, respectively. One participant in Part 2 experienced a treatment-emergent AE (ventricular extrasystoles) that led to discontinuation; it was considered unlikely that the event was related to study treatment. One participant in Part 2 experienced rhabdomyolysis (high creatine kinase and high aspartate aminotransferase), which was considered mild and unlikely to be related to study treatment; the event resolved without treatment after 11 days.

## Discussion

The data from these studies demonstrate that JNJ-77242113 is a highly potent peptide that selectively targets IL-23R and inhibits IL-23 signaling. JNJ-77242113 binds to IL-23R with affinity in the single-digit picomolar range. In vitro, JNJ-77242113 inhibited IL-23–induced proximal signaling through IL-23R (STAT3 phosphorylation) and production of downstream effectors (e.g. IL-17A, IFNγ) in a concentration-dependent manner, at the pico- to low nanomolar concentrations tested. These effects of JNJ-77242113 were selective for IL-23R, with no apparent impact on IL-12 signaling; while IL-23–induced STAT3 phosphorylation was inhibited, IL-12–induced STAT4 phosphorylation was not. This is in contrast to the TYK2 inhibitor, deucravacitinib (BMS-986165), which inhibits both IL-12– and IL-23–dependent phosphorylation of STAT4 and STAT3, respectively, in addition to Type 1 IFN signaling^28^. Furthermore, even at a concentration 1000-fold higher than those measured in humans dosed with 1000 mg, JNJ-77242113 demonstrated minimal activity against a panel of 44 potential secondary targets, providing additional evidence that JNJ-77242113 is highly selective for IL-23R at pharmacodynamically active concentrations.

JNJ-77242113 demonstrated efficacy at low oral doses in a rat model of intestinal inflammation despite relatively low systemic exposures (Table S2). Additionally, orally administered JNJ-77242113 demonstrated pharmacodynamic activity in rat blood and in a model of skin inflammation. The high affinity of JNJ-77242113 for IL-23R and exquisite potency for inhibition of IL-23 signaling and downstream cytokine production, combined with the low in vivo expression of IL-23R, are all likely to contribute to this efficacy. Distribution of JNJ-77242113 may also impact its activity; nonclinical data in monkeys showed that JNJ-77242113 distributed freely to a variety of tissues (e.g. colon, ileum, and skin), compared to the limited tissue distribution reported for monoclonal antibodies^29^. In both rat inflammation models, tissue-specific signs of inflammation (e.g. colon weight/length ratio or ear skin thickness) were alleviated in response to oral JNJ-77242113. In addition, IL-23–induced signaling was inhibited in blood and skin of rats dosed with oral JNJ-77242113. Of note, in rat skin, the extent to which oral JNJ-77242113 inhibited IL-23–induced IL-17A,-17F and -22 expression was comparable to that of an IL-23 monoclonal antibody given via intraperitoneal injection.

The activity of JNJ-77242113 observed in vitro in human immune cells and in preclinical models translated to pharmacodynamic activity in healthy volunteers. In this first-in-human study, JNJ-77242113 demonstrated dose-proportional pharmacokinetics across a 100-fold dose range. Even in the presence of supra-physiological levels of IL-23 stimulation, robust, dose-dependent inhibition of IFNγ production was observed in ex vivo assays of whole blood from participants who received JNJ-77242113. While systemic pharmacodynamic activity of JNJ-77242113 was observed in blood from healthy volunteers, it is unknown how this activity will translate to efficacy in different disease states as unique dose regimens are required for currently approved anti–IL-23 antibodies in psoriasis and psoriatic arthritis compared to Crohn’s disease^3,30–32^. Future clinical studies of JNJ-77242113 are needed to evaluate dose- and exposure-dependent efficacy in different patient populations to determine appropriate doses for specific diseases.

In healthy volunteers, JNJ-77242113 was well tolerated following single- or multiple-dose administration. AEs were mild or moderate and no dose-related AE trends were identified. Beyond the characteristics of JNJ-77242113, monoclonal antibodies targeting IL-23 have demonstrated that they are well tolerated, supporting the viability of selectively targeting the IL-23 pathway^5,22–25^. Ultimately, larger clinical studies will be needed to confirm the safety profile of JNJ-77242113; these studies are ongoing. Of note, in a recent phase 2 study of patients with moderate-to-severe psoriasis, JNJ-77242113 met its primary clinical efficacy endpoint and had a safety profile similar to placebo^26^.

In summary, these findings support further development of JNJ-77242113 as a first-in-class, potent, and selective oral therapy to target immune-mediated inflammatory diseases driven by dysregulated IL-23 signaling.

## Methods

### In vitro pharmacology

#### Recombinant protein expression

The p19 chain (IL23p19.1-189.GS.6His) and the p40 chain (IL23p40.1-328) were produced by secreted co-expression in human embryonic kidney (HEK)293-6E cells and purified by Ni affinity chromatography and polished with size exclusion chromatography (SEC).

Biotinylated recombinant human IL23R (hIL23R^biot^, hGH.IL23R.24-330.24-330.GS.Avi.TEV.8His) and rat IL23R (rIL23R^biot^, hGH.ratIL23R.24-317.GS.Avi.TEV.8His) were produced by secreted expression in HEK cells using a human growth hormone signal sequence. Both proteins were purified similarly from clarified media using Ni affinity chromatography then incubated with TurboTEV (Accelagen) and TurboBiotinylase (Accelagen) overnight at 4 °C. The proteins were further purified using subtractive Ni affinity, ion exchange and finally polished by SEC.

#### Surface plasmon resonance

Binding affinity and kinetics of JNJ-77242113 to the extracellular domain of human and rat IL-23R were evaluated using SPR. A streptavidin coated SPR chip (Cytiva [previously GE Healthcare]; Marlborough, MA) activated with 50 mM N-hydroxysuccinimide (NHS)/250 mM 1-ethyl-3-(3-dimethylaminopropyl)carbodimide (EDC) (Cytiva) was used to capture ~ 1200 RU biotinylated human IL-23R and the chip surface was deactivated with 1 M ethanolamine (Cytiva). A streptavidin-coated SPR chip was used to capture ~ 2500 RU biotinylated rat IL-23R (Cytiva). The unbound chip surface was blocked with 100 µM Amine-PEG2-Biotin (ThermoFisher Scientific; Waltham, MA) at 37 °C. JNJ-77242113 was injected over the immobilized IL-23R at multiple concentrations in run buffer (PBS containing 0.05% P20 [Cytiva] and 5% DMSO [EMD Millipore]) at 37 °C. Association and dissociation times were 280 s and 6000 s, respectively, for human IL-23R and 150 s and 2000 s, respectively, for rat IL-23R. All experiments were conducted on a Biacore 8 K + (Cytiva) in parallel kinetics mode and data fit to a 1:1 binding model in the Biacore Insight Evaluation software (Cytiva).

#### Human PBMC pSTAT assay

Human PBMCs (AllCells; Alameda, CA) were cultured for 4 days at 37 °C in 5% CO_2_ on anti-CD3–coated flasks in medium supplemented with 100 ng/mL IL-1β (BioLegend; San Diego, CA). To distinguish between the IL-23 and IL-12 signaling cascades, which share the IL-12Rβ1 receptor subunit, JNJ-77242113 was tested for its ability to inhibit IL-23–induced STAT3 phosphorylation and IL-12–induced STAT4 phosphorylation. PBMCs were stimulated with 5 ng/mL rhIL-23 (Janssen; internally produced) or rhIL-12 (R&D Systems; Minneapolis, MN) for 30 min and then lysed for measurement of phosphorylated STAT3 and STAT4 proteins using a phospho-STAT kit (Meso Scale Discovery [MSD]; Rockville, MD).

#### NK cell-based cytokine production assay

Human NK cells were enriched from PBMCs (Stanford Blood Center; Palo Alto, CA) using a magnetic enrichment kit (Stem Cell Technologies; Vancouver, Canada) and then cultured in medium supplemented with 25 ng/mL rhIL-2 (R&D Systems) for 7 days at 37 °C in 5% CO_2_. IL-2–activated cells were then stimulated with 10 ng/mL rhIL-18 (R&D Systems) and 3 ng/mL rhIL-23 (ThermoFisher Scientific) in the presence of increasing concentrations of JNJ-77242113 for 20–24 h at 37 °C in 5% CO_2_. Cell culture supernatants were collected and secreted IFNγ was measured using a Quantikine enzyme-linked immunosorbent assays (ELISA) kit (R&D Systems). IL-23–stimulated IFNγ concentrations were plotted against log-transformed peptide concentrations and a four-parameter model was used to determine the IC_50_ value for JNJ-77242113.

#### Screen for potential secondary targets

In vitro selectivity of JNJ-77242113 (10 μM) was evaluated at Eurofins Cerep (Celle-Lévescault, France), using the SafetyScreen44™ Panel (Eurofins).

### In vivo pharmacology

#### Ethics

Animal studies were conducted in accordance with animal protocols and procedures approved by the Institutional Animal Care and Use Committee and aligned with ARRIVE guidelines.

#### Rat TNBS-induced colitis model

Male Sprague–Dawley rats (Envigo; Frederick, MD) were administered either vehicle (water) or JNJ-77242113 via oral gavage three times per day at total daily doses of 0.03, 0.1, 0.3, 1, 3, and 10 mg/kg/day on Day -2 through Day 6. Rats were deprived of food for 24 h prior to induction of colitis by intrarectal installation of TNBS 48 mg/kg (Sigma Aldrich; Raleigh, NC) in 50% ethanol on Day 0. All dose groups (n = approximately 10/group; up to 3 separate experiments) received TNBS, except negative controls, which received only water (n = 4). Rats were weighed daily. Euthanasia by overexposure to isoflurane occurred on Day 7. Following euthanasia, blood was collected retro-orbitally into serum separator microtainer tubes (Becton Dickinson; Franklin Lakes, NJ), centrifuged (2–3 min at 4 °C, 14,000 RPM), and stored at − 80 °C. Additionally, the whole colon was excised, measured, and photographed, and then cut open to allow for collection of the contents, which were weighed and flash frozen. After removal of the contents, colons were weighed and flash frozen. JNJ-77242113 concentrations in colon tissue and colon contents were evaluated by liquid chromatography tandem mass spectrophotometry (LC–MS/MS). Colon weight/length ratio was calculated.

#### Rat IL-23–induced skin inflammation model

Female Sprague–Dawley rats (Envigo) were administered either vehicle (PBS; n = 5 per group in 3 experiments) or JNJ-77242113 (1, 3, 10, or 30 mg/kg/day) via oral gavage twice daily (n = 10 per dose group in up to 3 experiments), beginning the day prior to IL-23 injection (Day -1) through Day 3. Other groups received anti–IL-23p19 antibody (Santa Cruz Biotechnology; Catalog #sc-271279LS) or isotype antibody via intraperitoneal injection on Days -1 and 3, as additional controls. On Day 0 through Day 3, rats were anesthetized with isoflurane and injected with 1.5 μg of recombinant rat IL-23 (R&D Systems) in 20 μL PBS, intradermally, in the right ear; controls received an equivalent volume of PBS. Body weight was recorded daily. Thickness of the right (IL-23 injected) ear was measured to the nearest 0.05 mm using calipers (Kroeplin GmbH; Schlüchtern, Germany). Animals were euthanized by CO_2_ asphyxiation on Day 4. Blood was collected via cardiac puncture, deposited into K_2_EDTA tubes, and centrifuged at 2000 × *g* for 20 min. Plasma was collected into 5% volume protease inhibitor and stored at − 80 °C until bioanalysis by LC–MS. For gene expression analyses (IL-22, IL-17A, and IL-17F), the right ear was excised, flash frozen, homogenized, and reverse transcription polymerase chain reaction (RT-PCR) was performed using a TaqMan gene expression array (ThermoFisher Scientific).

### Ex vivo pharmacodynamic analyses

#### IL-23–stimulated IL-17A production in rat blood

Female, 7-week-old Sprague–Dawley rats (Charles River Laboratories; Hollister, CA) were orally dosed with JNJ-77242113 (0.03, 0.1, 0.3, 1, 3, 10, 30, or 100 mg/kg/day; n =  ~ 6 per group; up to 3 separate experiments) or vehicle (water). Two or six hours after dosing, animals were euthanized by CO_2_ asphyxiation and blood was collected via closed cardiac puncture into heparinized vacutainer tubes. Blood samples were diluted in pre-warmed Roswell Park Memorial Institute (RPMI)-1640 with glutamine and N-2-hydroxyethylpiperazine-N’-2-ethanesulfonic acid (HEPES) at a 1:4 ratio of blood:medium. Blood was mixed and pipetted into assay plates and stimulated with recombinant rat IL-23 (R&D Systems; final concentration of 20 ng/mL) plus IL-1β (R&D Systems; final concentration of 4 ng/mL) or IL-1β alone. Assay plates were incubated at 37 °C in 5% CO_2_ for ~ 24 h, centrifuged at 1300 rpm for 6 min at room temperature, and cell supernatants were collected. IL-17A was measured using a rat IL-17A ELISA kit (Abcam; Cambridge, UK). A similar in vitro assay was performed in rat blood with direct addition of a range of concentrations of JNJ-77242113, to determine the in vitro IC_50_ value for inhibition of IL-23–induced IL-17A production.

#### IFNγ production in donor whole blood

Whole blood from healthy donors or patients with psoriasis was diluted with pre-warmed RPMI medium to achieve a final ratio of 1:2 (blood:medium), treated in vitro for 30 min with titrated JNJ-77242113 and then stimulated with rhIL-2 (10 ng/mL) and rhIL-18 (20 ng/mL) with or without rhIL-23 (2 ng/mL) for 24 h. IFNγ levels were quantified using a Human IFNγ Kit (MSD).

### Phase 1 clinical trial

This study was conducted under a protocol reviewed and approved by the Alfred Hospital Ethics Committee. This study was conducted in accordance with the principles of the Declaration of Helsinki and with the National Health and Medical Research Council National Statement on Ethical Conduct in Human Research. The conduct of the study was in accordance with the Notes for Guidance on Good Clinical Practice established from the International Conference on Harmonization guidelines and adopted by the Australian Therapeutics Goods Administration. All participants provided written, informed consent.

The purpose of the first-in-human, single-center, randomized, double-blind, placebo-controlled study was to determine the safety, tolerability, pharmacokinetics, and pharmacodynamics of JNJ-77242113 in healthy participants. Participants enrolled in Part 1 (SAD cohorts) received a single oral dose of JNJ-77242113 or placebo on Day 1 following an overnight fast of approximately 10 h. Participants enrolled in Part 2 (MAD cohorts) received oral JNJ-77242113 or placebo once daily for 10 consecutive days; on Days 1 through 10, the study drug was administered following a 10-h fast. AEs were coded using the Medical Dictionary for Regulatory Activities (Version 23.1).

Whole blood samples were collected in TruCulture Tubes containing 10 ng/mL IL-2 and 20 ng/mL IL-18 with or without additional IL-23 (0.5 ng/mL, R&D Systems/Biotechne). JNJ-77242113 plasma concentrations were analyzed using a validated LC–MS/MS method. IFNγ was quantified via the V-Plex-plus human IFNγ kit (MSD).

### Statistical analyses

#### In vitro assays

Means ± SD values were calculated for pharmacological parameters based on results from ≥ 3 experimental runs (unless otherwise indicated). IC_50_ values for JNJ-77242113 were calculated in GraphPad Prism using non-linear regression (curve fit) – log[inhibitor] versus response.

For human whole blood assays, data from all assay runs were compiled together and a four-parameter logistic regression model with fixed and random (mixed) effects model was applied. A log_10_-transformation was performed on the IFNγ levels of all studies before running the model, and then back transformed to determine the peptide IC_50_ mean, median, and individual donor values.

#### Rat TNBS-induced colitis model

Body weight was analyzed by longitudinal regression analysis and Tukey–Kramer’s test approach to compare treatment effects of unbalanced data. For colon weight/length ratios, the estimated treatment effect for each treatment group was compared against the TNBS group. Colon weight/length ratio data were transformed to fit normality as determined by the Box-Cox method and analyzed by robust regression analysis with treatment group as a factor and M-estimation with bi-square weight function.

#### Rat IL-23–induced skin inflammation model

For expression analyses, data from 3 experiments were combined and treatment groups were compared with controls using a robust regression analysis. Ear thickness measurements were analyzed using generalized least squared models. Post-hoc statistical tests were adjusted using the false discovery rate with a threshold of *P* < 0.05 for statistical significance.

#### In vitro and ex vivo rat blood analyses

In experiments in which blood was treated with JNJ-77242113 in vitro, IL-17A levels were plotted versus log-transformed peptide concentrations in GraphPad Prism and the IC_50_ value was calculated using nonlinear regression (curve fit) – log[inhibitor] versus response (three parameters) – least squares regression. To estimate the ex vivo IC_50_ value for orally dosed JNJ-77242113, IL-17A levels were plotted versus log-transformed plasma peptide levels for individual (dosed) animals and IC_50_ values were calculated in GraphPad Prism using nonlinear regression (curve fit) – log[inhibitor] versus response (four parameters) – robust regression. Comparative statistics were performed using a one-way Analysis of Variance (ANOVA). Post hoc statistical tests were adjusted using either Dunnett’s multiple comparisons to compare each dose group to the vehicle or Sidak’s multiple comparisons for selected comparisons with a statistically significant p-value threshold of *P* < 0.05.

#### First-in human study

Mean concentration–time profiles for each treatment/dose group were presented graphically. Descriptive statistics were used to describe demographic and safety data.

### Supplementary Information

Supplementary Information.

## Data Availability

The data sharing policy of Janssen Pharmaceutical Companies of Johnson & Johnson is available at http://www.janssen.com/clinical-trials/transparency. The data supporting the findings of this study may be obtained from the authors upon reasonable request.
