# Supplementary material for: JNJ-77242113, a highly potent, selective peptide targeting the IL-23 receptor, provides robust IL-23 pathway inhibition upon oral dosing in rats and humans
Source: Sci Rep. 2024 Jul 30;14:17515. doi: 10.1038/s41598-024-67371-5 (PMC11289455; doi:10.1038/s41598-024-67371-5)
Supplement: Supplementary file 1 — Supplementary Information. [file 41598_2024_67371_MOESM1_ESM.docx]

**JNJ-77242113, a Highly Potent, Selective Peptide Targeting the IL-23 Receptor, Provides Robust IL-23 Pathway Inhibition Upon Oral Dosing in Rats and Humans**

**Supplementary Material**

**Table S1. Selectivity profiling of potential secondary targets of JNJ-77242113.** Values shown are percent inhibition of control ligand binding in the presence of 10 μM JNJ-77242113.

|  | **Percent inhibition of control ligand binding** | | |
| --- | --- | --- | --- |
| **Secondary target** | **1^st^ run** | **2^nd^ run** | **Mean** |
| A_2A_ | -8.7 | -6.2 | -7.5 |
| α_1A_ | -4.6 | 5.5 | 0.5 |
| α_2A_ | 0.4 | -2.6 | -1.1 |
| β_1_ | -5.1 | 7.7 | 1.3 |
| β_2_ | -8.2 | -0.6 | -4.4 |
| BZD | -6.4 | -7.6 | -7.0 |
| CB_1_ | 2.5 | -6.9 | -2.2 |
| CB_2_ | -4.9 | -5.8 | -5.4 |
| CCK_1_ (CCK_A_) | -29.4 | -12.3 | -20.8 |
| D_1_ | 2.2 | 3.7 | 2.9 |
| D_2S_ | -6.6 | -6.6 | -6.6 |
| ET_A_ | -31.3 | -12.9 | -22.1 |
| NMDA | 7.0 | 5.5 | 6.3 |
| H_1_ | -8.4 | -9.8 | -9.1 |
| H_2_ | -26.6 | -22.0 | -24.3 |
| MAO-A | 12.0 | 10.3 | 11.2 |
| M_1_ | -4.1 | 3.3 | -0.4 |
| M_2_ | 0.3 | 2.0 | 1.2 |
| M_3_ | -10.5 | -5.2 | -7.8 |
| N neuronal α4β2 | -10.4 | -7.5 | -8.9 |
| δ (DOP) | -7.8 | 0.4 | -3.7 |
| Kappa (KOP) | -2.5 | 6.8 | 2.1 |
| μ (MOP) | 5.1 | -4.8 | 0.1 |
| 5-HT_1A_ | -5.6 | 2.8 | -1.4 |
| 5-HT_1B_ | 0.0 | -9.8 | -4.9 |
| 5-HT_2A_ | 7.0 | 3.3 | 5.1 |
| 5-HT_2B_ | -19.6 | -6.7 | -13.1 |
| 5-HT_3_ | 0.7 | 3.4 | 2.1 |
| GR | 3.5 | -0.8 | 1.3 |
| AR | -5.6 | -3.7 | -4.7 |
| V_1a_ | 4.8 | 6.4 | 5.6 |
| Ca^2+^ channel (L, dihydropyridine site) | -8.7 | -24.0 | -16.3 |
| Potassium channel hERG | -13.0 | -11.9 | -12.4 |
| K_V_ channel | -3.8 | 0.5 | -1.6 |
| Na^+^ channel (site 2) | 0.0 | -3.6 | -1.8 |
| Norepinephrine transporter | -1.3 | -0.6 | -1.0 |
| Dopamine transporter | -3.6 | -4.4 | -4.0 |
| 5-HT transporter | -19.9 | 1.4 | -9.2 |
| COX1 | -18.8 | -37.6 | -28.2 |
| COX2 | 7.8 | -0.9 | 3.4 |
| PDE3A | -30.6 | -44.7 | -37.6 |
| PDE4D2 | -22.6 | -11.7 | -17.1 |
| Lck kinase | 8.6 | -2.3 | 3.1 |
| acetylcholinesterase | 9.8 | 2.2 | 6.0 |

A_2A_, adenosine 2A receptor; α_1A_, alpha-1A adrenergic receptor; α_2A_, alpha-2A adrenergic receptor; β_1_, beta-1 adrenergic receptor; β_2_, beta-2 adrenergic receptor; BZD, benzodiazepine receptor; CB_1_, cannabinoid receptor type 1; CB_2_, cannabinoid receptor type 2; CCK1, cholecystokinin 1 receptor; D_1_, dopamine D1 receptor; D_2S_, short dopamine D2 receptor; ET_A_, endothelin receptor subtype A; NMDA, N-methyl-D-aspartate receptor; H_1_, histamine H1 receptor; H_2_, histamine H2 receptor; MAO-A, monoamine oxidase A; M_1_, muscarinic acetylcholine receptor M1; M_2_, muscarinic acetylcholine receptor M2; M_3_, muscarinic acetylcholine receptor M3; N neuronal α4β2, neuronal alpha-4-beta-2 nicotinic acetylcholine receptor; δ (DOP), delta opioid receptor; *κ* (KOP), kappa opioid receptor; μ (MOP), mu opioid receptor; 5-HT_1A_, serotonin 1A receptor; 5-HT_1B_, serotonin 1B receptor; 5-HT_2A_, serotonin 2A receptor; 5-HT_2B_, serotonin 2B receptor; 5-HT_3_, serotonin 3 receptor; GR, glucocorticoid receptor; AR, androgen receptor; V_1a_, vasopressin receptor 1A; hERG, human ether-a-go-go related gene; K_V_, voltage-gated potassium channel; COX1, cytochrome c oxidase subunit 1; COX2, cytochrome c oxidase subunit 2; PDE3A, phosphodiesterase 3A; PDE4D2, cAMP-specific 3’,5’-cyclic phosphodiesterase 4D2; Lck, lymphocyte-specific protein tyrosine kinase.

**Table S2. Rat tissue pharmacokinetic parameters after oral administration of 10 mg/kg JNJ-77242113.** JNJ-77242113 (10 mg/kg) in 50 mM phosphate buffer (pH 7.4) was dosed orally to male Sprague-Dawley rats (n=4/timepoint). Plasma, small intestine, colon, and colon contents were sampled at terminal time points. Tissues and content were homogenized in buffer (1:1 PBS:ethanol with EDTA-free protease inhibitor tablets [Roche]). All samples were analyzed using LC-MS/MS. The pharmacokinetic results based on mean concentration profiles showed high exposure in intestinal tissues relative to plasma exposure.

| **Tissue** | **T_max_ (hr)** | **C_max_ (ng/mL or ng/g)^a^** | **AUC_last_ (ng·hr/mL or ng·hr/g)^a^** |
| --- | --- | --- | --- |
| Systemic plasma | 3 | 6.49 | 23.1 |
| SI | 1 | 2,090 | 4,950 |
| SI mucus | 1 | 58,500 | 105,000 |
| SI mucosa | 1 | 12,800 | 27,100 |
| Colon | 6 | 1,380 | 5,670 |
| Colon content | 6 | 371,000 | 1,700,000 |

^a^Systemic plasma C_max_ and AUC_last_ are expressed in ng/mL or ng·hr/mL, respectively; intestinal tissue concentrations and AUC_last_ are reported in ng/g or ng·hr/g, respectively.

AUC, area under the curve; C_max_, maximal concentration; LC-MS/MS, liquid chromatography tandem mass spectrophotometry; SI, small intestines; T_max_, time to maximal concentration.

**Table S3. Exposure in colon tissue and colon contents in individual TNBS studies, for which combined data is shown in Figures 2A and 2B.** Tissues and content were homogenized in buffer (1:1 PBS:ethanol with EDTA-free protease inhibitor tablets [Roche]). All samples were analyzed using an LC-MS/MS method (LLOQ=2.6 nM).

| **Treatment Group** | **Colon tissue (nM)** | | | **Colon content (nM)** | | |
| --- | --- | --- | --- | --- | --- | --- |
|  | **Study 1** | **Study 2** | **Study 3** | **Study 1** | **Study 2** | **Study 3** |
| JNJ‑77242113 0.03 mg/kg/day | - | BQL | - | - | 90.1 | - |
| JNJ‑77242113 0.1 mg/kg/day | 12.8 | 16.8 | BQL | 410 | 310 | 299 |
| JNJ‑77242113 0.3 mg/kg/day | 25.4 | 47.5 | 23.4 | 973 | 1050 | 1680 |
| JNJ‑77242113 1 mg/kg/day | 84.3 | 168 | 83.2 | 3340 | 3920 | 3190 |
| JNJ‑77242113 3 mg/kg/day | 333 | 468 | 268 | 9210 | 11700 | 11000 |
| JNJ‑77242113 10 mg/kg/day | - | 1410 | - | - | 38700 | - |

BQL, below quantification limit; LC-MS/MS, liquid chromatography tandem mass spectrophotometry; LLOQ, lower limit of quantitation; PBS, phosphate buffered saline; TNBS, trinitrobenzene sulfonic acid.

**Table S4. Demographics and baseline characteristics for participants in the Phase 1 study.**

|  | **Part 1 (SAD) (n=39)** | **Part 2 (MAD) (n=56)** |
| --- | --- | --- |
| Mean (SD) age, y | 27.0 (8.8) | 30.6 (8.8) |
| Male, n (%) | 39 (100) | 56 (100) |
| Race, n (%) |  |  |
| Asian | 17 (44) | 16 (29) |
| Black or African American | 2 (5) | 0 |
| White | 20 (51) | 37 (66) |
| Other | 0 | 3 (5) |
| Ethnicity, n (%) |  |  |
| Hispanic or Latino | 2 (5) | 5 (9) |
| Not Hispanic or Latino | 37 (95) | 45 (80) |
| Not reported | 0 | 2 (4) |
| Unknown | 0 | 4 (7) |
| Mean (SD) weight, kg | 76.0 (11.9) | 78.7 (12.5) |
| Mean (SD) height, cm | 177.9 (5.8) | 176.7 (7.3) |
| Mean (SD) BMI, kg/m^2^ | 23.9 (3.0) | 25.2 (3.5) |

BMI, body mass index; MAD, multiple ascending dose; SAD, single ascending dose; SD, standard deviation.

**Figure S1.** Representative sensorgrams displaying concentration-dependent and saturable binding of JNJ-77242113 to hIL-23R (**A**) and rIL-23R (**B**). Data is represented by the colored lines; black lines represent the 1:1 binding model. hIL-23R, human interleukin-23 receptor; rIL-23R, rat interleukin-23 receptor; RU, resonance unit.

**A.**

**
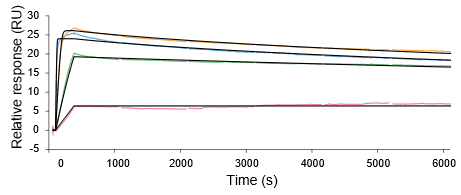
**

**B.**

**
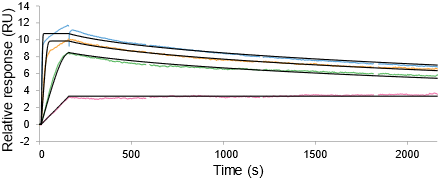
**

**Figure S2. JNJ-77242113 inhibited concentration-dependent induction of STAT3 phosphorylation by IL-23 in human DB cells.** DB cells were treated for 30 minutes with increasing concentrations of JNJ-77242113 and stimulated with rhIL-23 (Humanzyme; Chicago, IL). Controls included unstimulated cells and cells stimulated with IL-23 in the absence of JNJ-77242113. Cells were treated in accordance with the manufacturer’s Phospho-STAT3 (Tyr705) Homogeneous Time-Resolved Fluorescence assay protocol. The fluorescence emissions at 665 nm and 620 nm were measured. Homogeneous time-resolved fluorescence ratios expressed as normalized values (% response) from two assay plates were plotted as a function of log-transformed IL-23 concentrations. The K_d_ and mode of binding between JNJ-77242113 and IL-23R were evaluated using an assay in which pSTAT3 was measured in the presence of increasing concentrations of IL-23 stimulus and fixed concentrations of JNJ-77242113. Schild regression was analyzed using Gaddum/Schild EC_50_ shift in GraphPad Prism 8.3.0 software, generating a Schild slope of 1.09. A K_d_ of JNJ-77242113 for IL-23R was calculated as 15.1 pM. For the IL-23–titrated samples containing JNJ-77242113, each data point represents the mean for duplicated points. For the IL-23–titrated samples without JNJ-77242113, each point represents the mean for quadruplicate points. Error bars denote standard deviations. DB, diffuse large-cell B-lymphoma; IL, interleukin; K_d_, equilibrium dissociation constant; STAT3, signal transducer and activator of transcription.
